# Effect of Cyclic Loading on the Fixture-Abutment Microgap in Short Implants Versus Standard Implants: An In Vitro Study

**DOI:** 10.1155/ijod/4723112

**Published:** 2024-12-27

**Authors:** Gholamreza Esfahanizadeh, Ezatollah Jalalian, Seyyede Niloufar Salehi, Mahsa Ghasemi, Shaghayegh Golalipour

**Affiliations:** ^1^Prosthodontics Department, Faculty of Dentistry, Tehran Medical Sciences of Azad University, Tehran, Iran; ^2^Faculty of Dentistry, Tehran Medical Sciences of Azad University, Tehran, Iran; ^3^Department of Prosthodontics, Faculty of Dentistry, Tehran Medical Sciences of Azad University, Tehran, Iran

**Keywords:** dental implant-abutment design, dental implants, mastication

## Abstract

**Objectives:** This study aimed to assess the effect of cyclic loading on the amount of fixture-abutment microgap in short implants compared to standard implants.

**Materials and Methods:** This in vitro experimental study was conducted on two groups of short and standard implants (*n* = 10). The microgap at the fixture-abutment interface was measured under a light microscope at ×75 magnification. The implants were mounted in an acrylic resin to simulate the jawbone. They were then subjected to cyclic loading by applying 75 N load with 1 Hz frequency along the longitudinal axis of each implant (perpendicular to the abutment surface). After 500,000 cycles, corresponding to 20 months of mastication in the oral environment, the implants were removed from the acrylic resin, and the microgap at the fixture-abutment interface was measured again under a stereomicroscope by a blinded examiner. Data were then analyzed by *t*-test using SPSS version 22 (*α* = 0.05).

**Results:** The mean microgap was 13.59 ± 3.80 µm in the standard and 20.41 ± 11.30 µm in the short implants before cyclic loading (*p*=0.087). These values changed to 15.22 ± 5.44 and 24.53 ± 21.85 µm, respectively, after cyclic loading. No significant difference was noted in the amount of microgap between the standard and short implants after cyclic loading (*p*=0.222).

**Conclusion:** Cyclic loading increased the amount of fixture-abutment microgap in both the standard and short implants. However, the difference in this respect was not significant between the two implant lengths. Thus, short implants could be reliably used in patients with limitations for surgery to restore function and esthetics.

## 1. Introduction

A dental implant treatment plan should include the ideal size of the implant, which is determined based on biomechanical and esthetic considerations [1]. In the past, implant size used to be determined merely based on the available bone volume (height, width, and length of bone). The surgeons used to place longer implants in the anterior region and shorter implants (or cantilever prostheses) in the posterior region due to the limitations imposed by the mandibular canal and the maxillary sinus. Also, implant diameter would be selected at the time of surgery, depending on the available bone width, and implants with 4 mm diameter would be placed in most cases [2].

Friberg et al. [3] were the first to introduce short implants for the reconstruction of severely atrophic edentulous mandibles in 2000. A short implant refers to a dental implant with a fixture length shorter than 7 mm [4]. Short implants are more commonly used in areas adjacent to anatomical landmarks and critical structures as well as in the posterior areas with limited bone height [4]. Minimum bone height is often recorded in the posterior areas due to the extension of the maxillary sinus after tooth loss, and the presence of the mandibular canal 10 mm or higher above the inferior border of the mandible [1].

Studies have shown that in many cases, the available bone height in the posterior regions is often insufficient for standard length implants, as reported in the radiographic study on 431 partially edentulous patients, indicated that the available bone height in the posterior region was ≥6 mm in 38% of the maxillae and 50% of the mandibles [5]. Also, it has been reported that the posterior areas of the jaw in completely edentulous patients have >6 mm bone height in less than 20% of the cases. Thus, it necessitates the use of short implants unless extensive ridge augmentation procedures (e.g., maxillary sinus floor augmentation, onlay bone grafts, and nerve transposition) are performed [5].

The reported failure rates of short implants have been variable; however, recent studies have shown improved prognosis of short implants over the years [4, 6–11]. Goodacre, Kan, and Rungcharassaeng [12] in their review study showed a higher failure rate of implants shorter than 10 mm. They reported the failure rate of shorter implants to be 15%, compared with 3% failure rate of longer implants. The failure rate was higher for implants shorter than 10 mm placed in the posterior areas in partially edentulous patients. Less than 50% of clinical reports showed a survival rate of over 90%, and over 50% of the reports indicated a failure rate of over 19.7% for short implants. It should be noted that most of the failures were not related to the surgical procedure or failed osseointegration. Failure of short implants mainly occurred following their prosthetic loading. In other words, the success of implant surgery was not influenced by the implant length. However, after loading, a rise in failure rate was noticed due to early loading, particularly in the first 6–18 months. Higher failure rate of short implants after loading, compared with longer implants, may be explained by four biomechanical reasons [13]: (I) smaller surface area of short implants for distribution of occlusal loads, (II) higher masticatory forces in the posterior region, (III) lower bone density in the posterior region, and (IV) increased crown height.

Microgap at the implant-abutment interface is another problem that can cause implant failure [14]. Microgap refers to the distance between the abutment and the fixture. Microgap formation at the implant-abutment interface has been strongly linked to both biological and mechanical complications that can lead to implant failure. The presence of a microgap can act as a site for bacterial colonization, leading to peri-implantitis and subsequent bone loss [15, 16].

Moreover, studies have shown that microgap can result in mechanical complications, such as micromovements and loosening of the implant-abutment connection, which ultimately compromise the stability of the implant [14, 17]. This compromised stability can accelerate prosthetic failure and increase the risk of implant failure, particularly in cases with early loading [13]. Recent studies have further validated this link, highlighting the importance of minimizing microgap to improve implant longevity [18, 19].

In addition to the risk of microleakage, microgap formation at the implant-abutment interface poses a significant threat to the prosthetic joint's structural integrity. A poorly sealed joint can experience micromovements under cyclic loading, which may lead to joint loosening, abutment fractures, and eventual implant failure [17]. These biomechanical challenges are exacerbated in the posterior region, where occlusal forces are greater, increasing the stress on the implant's prosthetic components [14, 20]. The prosthetic joint failure, driven by these forces, can further worsen complications, necessitating repair or replacement of the implant.

Prosthetic joint design also plays a significant role in the biomechanical performance of dental implants. In a previous study, they found that the connection type and cyclic loading did not significantly affect the microleakage; although the internal connection showed lower torque loss than the external connection design [21]. However, conical connection designs have been shown to offer superior resistance to microgap formation and leakage under oblique loading, compared to external hexagonal designs; reported that 90% of implants with conical connection showed leakage under 100 N load, while 80% of implants with external hexagonal connection showed leakage under 40 N load [22, 23]. This improved sealing and load distribution reduces the risk of joint failure, which is a common issue with external hexagonal designs under functional loading [24].

Despite the increasing use of short implants, the gap and micromovement of short implants under cyclic loading have been less commonly addressed in the literature. Thus, this study aimed to assess the effect of cyclic loading on the amount of abutment-fixture microgap in short implants compared with standard implants. The null hypothesis was that no significant difference would be found in the amount of microgap between the short and standard implants, and cyclic loading would have no significant effect on the amount of microgap at the implant-abutment interface.

## 2. Materials and Methods

### 2.1. Statistical Analysis

The sample size was calculated to be 10 in each group of short and standard implants, assuming the standard deviation of the microgap to be 1.8 µm, a mean difference of 3 units, *α* = 0.05, *β* = 0.2, and a study power of 80% [18]. This in vitro experimental study was conducted on two groups of short and standard implants (*n* = 10 from each).

**Figure 1:**
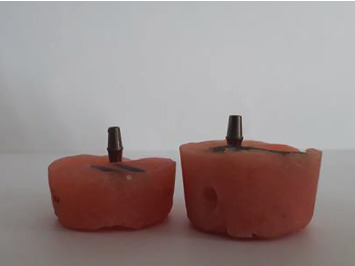
The dental implants were mounted in the acrylic molds in vertical position.

Acrylic resin was used to simulate the jawbone in this study. Molds with 13 mm height and 34 mm diameter were used to simulate low bone height in cases with bone resorption, and molds with 19 mm height and 34 mm diameter were used to simulate normal bone height [19]. Implants were mounted in acrylic molds filled with autopolymerizing acrylic resin (Acropars, Tehran, Iran). A surveyor (J.M. Ney Co., Bloomfield, CT, USA) was used to mount the implants in the molds in a completely vertical position (90° angle relative to the horizon; Figure 1). The resin blocks were then fixed in the jig and the abutments (Megagen, Korea) were tightened on the implants. The abutments were tightened to 30 N/cm using a digital torque meter (Lutron Electronic Enterprise Co., Taiwan) according to the manufacturer's instructions.

### 2.2. Implant Parameters

Grade 5 titanium root form implants (Megagen, Korea) with internal connection and hexagonal connector were used as bone level in this study. The short implants had 7 mm height and 4.5 mm diameter, while the standard implants had 10 mm height and 4.5 mm diameter. Straight abutments with 5.5 mm height and 4.5 mm diameter were used with 5.5 mm cuff height for the short and 2.5 mm cuff height for the standard implants.

After mounting the implants and placement of the abutments, the acrylic surface was marked to indicate the mesial, distal, buccal, and lingual surfaces, and then the microgap at the implant-abutment interface was measured under a stereomicroscope (SMZ800, Nikon, Japan) with ×75 magnification at four points at the mesial, distal, buccal, and lingual.

**Figure 2:**
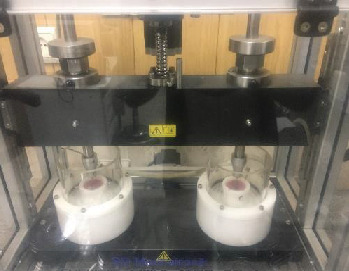
Simulating mastication forces using cyclic load machine.

Next, the specimens were subjected to cyclic loading (Cs-4 chewing simulator; Mechatronik, Germany) with 75 N force and 1 Hz frequency, applied along the longitudinal axis of the specimens (perpendicular to the abutment surface; Figure 2) [18, 25, 26].

**Figure 3:**
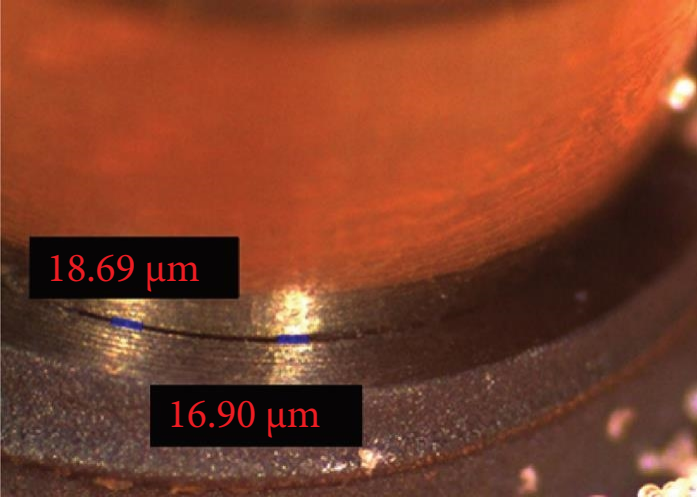
Measuring the microgap at the fixture-abutment interface under a stereomicroscope (×75 magnification) in the nonloaded condition, with the right number referring to standard implant and the left one referring to the short implant.

After applying 500,000 cycles corresponding to 6 months of mastication in the oral environment [25, 26]. The microgap at the implant-abutment interface was measured under a stereomicroscope (SMZ800, Nikon, Japan) with ×75 magnification at four points (mesial, distal, buccal, and lingual). The measurements were repeated by the same examiner, with an intraobserver agreement of one in a blind manner (Figure 3). The microscope was calibrated before each set of measurements to ensure precision. All the four points for each specimen were compared between case and control groups, before and after cycling loads. Then the case and control groups were compared were compared.

Data were analyzed using SPSS version 22. The Kolmogorov–Smirnov test was used to assess the normality of data distribution, which showed normal distribution of data. Thus, *t*-test was applied for the comparisons at 0.05 level of significance.

## 3. Results

Measurements of central dispersion for both the standard and short implant groups, before and after cyclic loading, are provided in the text and Table 1.

Regarding the round platform and the vertical force with 90° in all four points of buccal, mesial, distal, and lingual, the microgap is not affected depending on the location of different points; therefore, we measured the mean value of all four points for each sample, presenting a single microgap value for each specimen. The Kolmogorov–Smirnov test confirmed normality, allowing for a *t*-test comparison at a 0.05 significance level. The mean value and standard deviation of the gap before the cyclic loading in the standard implants and short implants were 13.59 ± 3.80 and 20.41 ± 11.30 µm, respectively. After the cyclic loading, the mean value and standard deviation for standard and short implants changed to 15.25 ± 5.44 and 24.53 ± 21.85 µm, respectively.

We performed a comparison of microgap measurements between the standard and short implant groups before and after cyclic loading using the *t*-test. The analysis reveals that although there was an increase in the microgap after cyclic loading, no significant difference was found between the two groups (*p*=0.087).

No visible signs of structural deformation, coupling loosening, or damage to the implant-abutment interface were observed in either group after cyclic loading. The interfaces remained intact throughout the testing process, and no fractures or visible wear were detected on the implants after applying the cyclic loading.

## 4. Discussion

Microgap is among the critical factors that can lead to implant failure. Due to the absence of the cushioning effect of the periodontal ligament around dental implants, its restoration and the supporting bone serve as a single functional unit. Thus, any mismatch between the implant components can cause irreversible damage to the surrounding bone [20]. Also, microgap leads to peri-implant mucositis which can adversely affect dental implant survival in the long-term [24].

Several factors influence the microgap, including the implant system, implant geometry, implant-abutment contact, and the applied torque used to tighten the abutment [27]. This study assessed the effect of cyclic axial loading on the fixture-abutment microgap in short implants compared to standard implants. While the results indicated no significant difference in microgap size between the two groups after loading, the short implants showed slightly higher, although not statistically significant, microgap values (15.25 µm versus 24.53 µm). These findings align with other studies that have reported microgaps as high as 49 µm [20, 28–30].

Several studies, including Lorenzoni et al. [31], have evaluated microleakage at the implant-abutment interface using different methodologies. Lorenzoni et al. [31] evaluated the microleakage through the implant-abutment interface of two hexagon dental implants (4.1 mm). They reported the presence of microgap at the implant-abutment interface in both implant types. Their results were in agreement with our findings despite different methodologies. They injected a red acid at the interface prior to torquing and immersed the fixture-abutment assembly in distilled water without cyclic loading. They assessed the microleakage by spectrophotometry. In the present study, cyclic loading was performed to simulate the masticatory forces since such forces create micromovements at the implant-abutment interface. The microgap also increases by opening and closing of the mouth, creating a pumping effect at the interface [31].

Nasser Mostofi et al. [32] evaluated the efficacy of GapSeal for the prevention of fluid leakage and the size of microgap in implants with internal connection. They measured the microgap to be 3.04 ± 0.54 µm in the control and 0.99 ± 0.39 µm in the test group. The amount of microgap in their study was within the acceptable range, similar to our study; although the cyclic loading protocol in their study was different from ours. GapSeal is a silicone gel that is applied to seal the interface. However, it undergoes degradation over time, questioning its successful clinical application.

He et al. [23], and Lee, Jo, and Noh [33] used finite element analysis to calculate the size of microgap. Lee, Jo, and Noh [33] reported that the amount of microgap in bone-level implants was 1.5–2 times the value in gingival-level implants, with a maximum microgap value of 22 µm, which was close to the value obtained in the present study. They concluded that conical connection had higher resistance against microgap formation at the implant-abutment interface. In another study by Lorussoet al. [22], it has been shown that conical connections can increase the stability comparing to hexagon connection after applying loading which can decrease microleakage significantly. Moreover, Scarano et al. [34] found that the microgap in the conical connection group was not present, on the contrary to the screw abutments.

In line with the present results, Lemos et al. [35] found no significant difference between the standard and short implants and reported that marginal bone loss, prosthetic failure, and complications of short implants were comparable to standard implants. Nonetheless, they recommended careful application of short implants especially in the posterior region.

Assessment of only one implant system, no simulation of other types of loads, such as destructive lateral forces, use of acrylic resin instead of bone, and not assessing short implants with different lengths, and the prosthesis configuration were among the limitations of this study. Future studies with longer follow-ups and better simulation of the oral clinical setting are recommended.

## 5. Conclusion

Cyclic loading increased the amount of fixture-abutment microgap in both the standard and short implants. However, the difference in this respect was not significant between the two implant lengths. Thus, short implants may be reliably used in patients with limitations for surgery to restore function and esthetics.

## Figures and Tables

**Table 1:** Measures of central dispersion for the amount of microgap in the short and standard implant groups before and after cyclic loading (µm).

Group	Time	Mean and standard deviation	Minimum	Maximum
Standard implant (*n* = 10)	Before cyclic loading	13.59 ± 3.80	5.45	17.04
	After cyclic loading	15.25 ± 5.44	8.62	23.37

Short implant (*n* = 10)	Before cyclic loading	20.41 ± 11.30	9.99	48.45
	After cyclic loading	21.85 ± 24.53	12.29	78.69

## Data Availability

The data are available in the manuscript and the table.
